# Effects of Antioxidant Treatment on Blast-Induced Brain Injury

**DOI:** 10.1371/journal.pone.0080138

**Published:** 2013-11-05

**Authors:** Xiaoping Du, Donald L. Ewert, Weihua Cheng, Matthew B. West, Jianzhong Lu, Wei Li, Robert A. Floyd, Richard D. Kopke

**Affiliations:** 1 Hough Ear Institute, Oklahoma City, Oklahoma, United States of America; 2 Oklahoma Medical Research Foundation, Oklahoma City, Oklahoma, United States of America; 3 Departments of Physiology and Otolaryngology, University of Oklahoma Health Sciences Center, Oklahoma City, Oklahoma, United States of America; Katholieke Universiteit Leuven, Belgium

## Abstract

Blast-induced traumatic brain injury has dramatically increased in combat troops in today’s military operations. We previously reported that antioxidant treatment can provide protection to the peripheral auditory end organ, the cochlea. In the present study, we examined biomarker expression in the brains of rats at different time points (3 hours to 21 days) after three successive 14 psi blast overpressure exposures to evaluate antioxidant treatment effects on blast-induced brain injury. Rats in the treatment groups received a combination of antioxidants (2,4-disulfonyl α-phenyl tertiary butyl nitrone and N-acetylcysteine) one hour after blast exposure and then twice a day for the following two days. The biomarkers examined included an oxidative stress marker (4-hydroxy-2-nonenal, 4-HNE), an immediate early gene (c-fos), a neural injury marker (glial fibrillary acidic protein, GFAP) and two axonal injury markers [amyloid beta (A4) precursor protein, APP, and 68 kDa neurofilament, NF-68]. The results demonstrate that blast exposure induced or up-regulated the following: 4-HNE production in the dorsal hippocampus commissure and the forceps major corpus callosum near the lateral ventricle; c-fos and GFAP expression in most regions of the brain, including the retrosplenial cortex, the hippocampus, the cochlear nucleus, and the inferior colliculus; and NF-68 and APP expression in the hippocampus, the auditory cortex, and the medial geniculate nucleus (MGN). Antioxidant treatment reduced the following: 4-HNE in the hippocampus and the forceps major corpus callosum, c-fos expression in the retrosplenial cortex, GFAP expression in the dorsal cochlear nucleus (DCN), and APP and NF-68 expression in the hippocampus, auditory cortex, and MGN. This preliminary study indicates that antioxidant treatment may provide therapeutic protection to the central auditory pathway (the DCN and MGN) and the non-auditory central nervous system (hippocampus and retrosplenial cortex), suggesting that these compounds have the potential to simultaneously treat blast-induced injuries in the brain and auditory system.

## Introduction

Blast-induced traumatic brain injury (bTBI) has dramatically increased in combat troops and civilians due to improvements in explosive devices employed in military conflicts and terrorist activities [1-3]. Blast exposure primarily affects gas-containing organs, such as the middle ear, lung, and gastrointestinal tract [4-9]. More recent evidence indicates that blast exposure also causes solid organ injury. Among these solid organs, the brain is very vulnerable to blast overpressure, due to the fact that shock waves can penetrate through the skull without significant change in amplitude and waveform [3,10,11]. bTBI causes acute and chronic neuropsychiatric sequelae both in human victims and in animal models [12,13]. Symptoms of mild TBI caused by blast include altered cognition, memory, motor coordination, and behavior [14-17]. Blast exposure can cause hemorrhage, edema, pseudoaneurysm formation, vasoconstriction, hypoperfusion in the brain, and disruption of the blood-brain barrier [3,18-25]. 

The mechanisms of blast-induced brain injury remain controversial [3,11,26]. Blast pressure waves may cause brain injury by directly transmitting blast energy into the brain and/or indirectly through dysfunction of the pulmonary and circulatory systems. Activation of the autonomic nervous system and the neuroendocrine-immune system may contribute to molecular changes and cellular injuries in the brain [19,27-35]. Cellular injuries include oxidative stress [18,22,28,36-38], astrocytic hyperplasia [11,17,23,34,39], diffuse axonal injury [14,17,20,21,32], inflammation [39-42], apoptosis [20,42,43], and neurodegeneration [12,20,23,42,43]. Degenerative processes, such as darkened atrophic dendrites and the accumulation of heavy subunits of neurofilament protein in neuronal soma, have been observed in the cerebral (i.e. temporal cortex) and cerebellar cortices and the hippocampus [33,35]. Long CNS axon tracts are particularly vulnerable to the effects of blast [44]. Cellular injury in the brain is blast dose-dependent. High-overpressure (> 10 MPa, 10 MPa ^≈^ 1450 psi) underwater shock wave exposure has been shown to result in hemorrhage, necrosis, and neuronal apoptosis mediated by a caspase-dependent pathway in the brain, while low-overpressure (1 MPa ^≈^ 145 psi) shock wave exposure has been shown to result in spindle-shaped changes in neurons and elongation of nuclei without marked neuronal injury [45,46]. Additionally, altered gene expression profiles, including factors responsible for cell death, inflammation, neurotransmission, and auditory function have been observed in the brain after blast exposure [43,47,48]. 

Oxidative stress and antioxidant depletion are associated with blast-induced brain and lung injuries [5,12,18,22,28,37,49]. Under these conditions, oxidative stress can lead to peroxidation of cellular and vascular structures, oxidation of cellular proteins, DNA damage, and inhibition of the mitochondrial electron transport chain, thus potentiating secondary damage in brain and lung tissues after these acute insults [50,51]. Consistent with these observations, brief pharmacological doses of antioxidant (vitamin E, vitamin C, or lipoic acid) loading have been shown to reduce blast-induced oxidative stress in the lung by increasing hemoglobin oxygenation and reducing lipid peroxidation [52,53]. The antioxidant N-acetylcysteine amide (NACA) significantly reduced pulmonary inflammation after blast exposure by blocking inflammatory chemokine mRNA expression in the lung [10]. These findings suggest that antioxidants have the potential to block the molecular cascades that are triggered by the blast exposure by opposing the oxidative stress conditions that lead to permanent brain damage and functional disability.

Previously, we demonstrated that N-acetylcysteine (NAC) plus 2,4-disulfonyl α-phenyl tertiary butyl nitrone (HPN-07) treatment can reduce both temporary and permanent hearing threshold shift and hair cell loss in the cochlea when administrated shortly after blast exposure [54]. Herein, we address the potential of this combinatorial treatment of antioxidants to also block damage within the CNS caused by blast overpressure. We chose a series of biomarkers that are expressed in brain tissue or cerebrospinal fluid after blast injury [20,32,55,56] to examine both the immediate (within a few hours) to intermediate (21 days after blast) effects of antioxidant treatment on bTBI. These biomarkers include 4-hydroxy-2-nonenal (4-HNE), a marker for oxidative stress; c-fos, a marker for neuronal activity; glial fibrillary acidic protein (GFAP), a marker for astrocyte activation; amyloid beta (A4) precursor protein (APP) [43,57] and 68 kDa neurofilament (NF-68), markers for axonal injury [58]; and caspase 3, a marker for apoptotic cell death. 

## Methods

### Animals, blast exposure, auditory brainstem responses (ABR), and distortion product otoacoustic emission (DPOAE) recording

All procedures regarding the use and handling of animals were reviewed and approved by the Oklahoma Medical Research Foundation (OMRF) Institutional Animal Care and Use Committee (IACUC) and the U.S. Department of the Navy Office of Naval Research. Male Long-Evans pigmented rats with body weights between 360 and 400g (Harlan Laboratories, Indianapolis, Indiana) were used in this study. The animals were housed and maintained in the animal care facility at OMRF. 

Blast exposure, administration of antioxidants, and details regarding measurement of ABR and DPOAE were detailed previously [54]. Only rats exposed to three blast overpressures at 14 psi were used in the present study. In brief, a blast simulator was custom built to generate blasts using compressed nitrogen against a plastic film. The body of the rat was protected by a holding tube and the top of its head was positioned perpendicular to the nozzle of the blast simulator. Each rat was exposed to 14 psi blasts repeated three times at 1.5-minute intervals under deep anesthesia (50 mg/kg of ketamine and 6 mg/kg of xylazine). Seventy-nine rats were exposed to this blast regimen. ABR thresholds and DPOAE levels were obtained for each rat prior to blast exposure and at 3 hours (H), 24H, 7 days (D), and 21D after exposure. ABR threshold shifts and DPOAE levels were recorded in a sound-attenuated, electrically shielded booth, using three stainless steel needle electrodes and a computerized Intelligent Hearing System (IHS) with Smart-EP software 3.96 (for ABR recording) or Smart OAE software 4.54 (for DPOAE recording). ABR thresholds for tonal bursts ranging from 2-16 kHz were determined. ABR threshold shifts were calculated by subtracting pre-blast exposure thresholds from post-blast exposure thresholds. DPOAE measurements were performed for pure tones ranging from 2-16 kHz. DPOAE level shifts were calculated by subtracting post-blast exposure levels from pre-blast exposure levels.

After blast exposure, rats were randomly assigned to either an antioxidant treatment group (B/T), which received NAC plus HPN-07 (see below), or a blast control group (B), which received an equivalent volume of saline. Each group of control and treated rats was designated for terminal analysis at 3H, 24H, 7D, or 21D, at which time ABR and DPOAE analyses were performed. Following the final ABR and DPOAE recording, all animals were euthanized and intracardially perfused with 4% paraformaldehyde in 0.1 M phosphate-buffered saline (PH 7.2) prior to harvesting brain and cochlear tissues for histological analyses of relevant biomarker levels and hair cell counts, respectively (detailed below). 

Following the final blast exposure, the outer ears of each rat were examined using a surgical microscope to assess the condition of the tympanic membrane. Eleven rats, each exhibiting dually-ruptured tympanic membranes, were excluded from the study. In total, 74 rats (6 in the normal control group, 68 in blast and blast plus treatment groups, with 6-7 rats in each group at each time point) were examined and analyzed in the present study. Rats in the normal control group (NC) did not receive blast exposure or drug treatment.

### Drug administration

NAC was purchased from Hospira, Inc. (Lake Forest, IL), and HPN-07 was provided at greater than 98.5% purity by APAC Pharmaceuticals LLC (Columbia, MD). The two antioxidants were combined and dissolved in a physiological saline solution to final concentrations of 60 mg/ml for each drug. Animals in the treatment group were injected intraperitoneally with a volume of the drug combination equivalent to 300 mg/kg of NAC and 300 mg/kg of HPN-07, beginning 1 hour after blast exposure and then twice-daily for the following two days. Rats in the blast control group received an equal volume of physiological saline solution according to the same schedule as the treatment group. According to the schedule, the 3H groups received one dose of drugs or saline, the 24H groups received three doses, and the 7D and 21D groups received five doses in total.

### Collection and sectioning of brains and brainstems

All tissue samples used in this study were collected and prepared for histological examination as described previously [54]. The brainstems ipsilateral to the ears exhibiting intact tympanic membranes and the corresponding cerebral tissues from the contralateral side were processed for histological study. Cochleae (total 108), brains (total 54), and brainstems (total 54) were removed and post-fixed in the same fixative (overnight for the cochleae and 1 week for the brain tissue) and then washed and stored in PBS at 4°C. The right cochlea from each animal was used for whole mount and TRITC-phalloidin staining for hair cell counting under a fluorescent microscope (Olympus BX51, Melville, NY). The percentage of missing hair cells was reported in a previous report [54]. The brain and brainstem from each animal was cryoprotected in 30% sucrose in PBS, embedded in Tissue-Tek (Sakura Finetek USA Inc. Torrance, CA), and serially sectioned in a coronal plane with a Thermo Cryotome (Thermo Fisher Scientific, Inc. Waltham, MA) at 18-20 µm. Every tenth section from each brainstem or brain was mounted onto a gelatin pre-coated slide (total of 10 slides for brainstem and of 20 slides for brain with 10-12 sections on each slide). 

### Biomarker expression analyses in the brain and brainstem

The biomarkers used in the present study included 4-HNE, c-fos, APP, GFAP, NF-68, and caspase 3. Most of these biomarkers have previously been reported to be expressed in brain tissue or cerebrospinal fluid after blast injury [20,32,55,56]. Anti-NeuN antibody was used to label neurons in the dorsal cochlear nucleus (DCN) 21 days after blast exposure to examine neuron loss in the DCN. The brain sections were washed with PBS, blocked in 1% bovine serum albumin (fraction V) and either 1% normal goat or horse serum in PBS, and permeabilized in 0.2% triton X-100 in PBS (PBS/T). The tissues were then incubated with either rabbit anti-c-fos IgG (1:100, Santa Cruz Biotechnology, Inc. Santa Cruz, CA), rabbit anti-GFAP IgG (1:500, EMD Millipore, Billerica, MA), rabbit anti-caspase 3 IgG (1:25, Santa Cruz Biotechnology, Inc. Santa Cruz, CA), rabbit anti-NeuN IgG (1:500, Chemicon International, Inc. Temecula, CA), or mouse anti-NF-68 IgG (1:200, Sigma, St. Louis, MO) overnight. After PBS/T washing, either biotinylated anti-rabbit IgG or anti-mouse IgG (1:200, Vector Laboratories, Inc. Burlingame, CA) was applied to the slides for 1 hour, and Vectastain ABC and DAB kits (Vector Laboratories, Inc. Burlingame, CA) were then used for the immunolabeling visualization. Immunopositive cells had a brown reaction product. Methyl green was used for nuclear counter-staining. C-fos expression was analyzed at all time points after blast exposure in the retrosplenial cortex (RC) and the DCN and at one time point (3H) in the rest of the brain regions [the auditory cortex (AC), the hippocampus, and the inferior colliculus (IC)]. Sections of rat brains from 7D and 21D groups were used for GFAP and NF-68 staining. Sections of brains from the normal control group were used as normal controls for each staining method. 

A set of brain sections (NC, 24H, and 7D groups) was used for fluorescence APP immunolabeling. After incubation with mouse anti-APP IgG (1:50, Lot # NG1850184, EMD Millipore, Billerica MA), the sections were incubated with Alexa Fluor^®^ 568 donkey anti-mouse IgG (1:1000, Life technologies, Co. Grand Island, NY). DAPI was used to label nuclei. Images were collected with a confocal microscope (Leica SP2 Confocal Microscope, Heidenberg, Germany). For 4-HNE fluorescence immunolabeling, a set of brain sections (NC, 3H, and 24H groups) were incubated with rabbit anti-4-HNE Michael adducts IgG (1:100, chemically Reduced, EMD Millipore, Billerica, MA). After washing with PBS, the sections were then incubated with Alexa Fluor^®^ 594 donkey anti-rabbit IgG (1:1000, Life technologies, Co. Grand Island, NY). DAPI was used to label nuclei. Fluorescence images were collected with an Olympus BX51 microscope (Melville, NY). Representative images were also collected with a Leica SP2 confocal microscope (Heidenberg, Germany). 

Blast- or noise-induced trauma have been shown to impair neurogenesis in the hippocampus [59,60]. To study whether blast exposure impaired neurogenesis in the brain in our experimental system, doublecortin antibody (goat anti-doublecortin IgG, 1:100, Santa Cruz Biotechnology, Inc. Santa Cruz, CA), a neurogenesis marker, was used to stain the brain sections of the NC and 21D groups.

To control for specificity of immunolabeling, negative controls were prepared for each staining by omitting the primary antibody incubation step. Positive control sections (Chemicon International, Temecula, CA) were used in caspase 3 staining analyses.

### Quantification of biomarker immunostaining

For quantification of immunostaining, images were collected from the RC (retrosplenial granular and dysgranular cortices), the DCN [the medial third (*medial*), the middle third (*middle*) and the lateral third (*lateral*)], the molecular layer of the dentate gyrus (MoDG), the *radiatum* and lacumosum molecular layers near the CA2 region of the hippocampus, the medial geniculate nucleus (MGN, dorsal and ventral), layers 2-5 of the primary AC, and the central nucleus of the IC. Images used for quantification were collected from all DCN sections (2-4 sections on each slide), 4-7 hippocampal sections, and 3-4 AC or IC sections from each rat. Identification of the nuclei or brain regions was guided by anatomical landmarks described within a pictorial atlas of the rat brain [61]. A modified two-dimensional quantification method was employed to count positive immunostained cells in these nuclei or regions [62,63]. A color camera (DP70) attached to an Olympus microscope (BX51) and DPController and DPManager programs (Olympus, Melville, NY) were used to obtain images. The distance between two adjacent sections on each slide was about 200 µm to ensure non-duplicate counting. The total number of positive cells within each image was counted using ImageJ software (National Institutes of Health), and cell density was calculated (cells/mm^2^) and statistically analyzed as detailed below. The density of doublecortin in the hippocampus was obtained by dividing the number of doublecortin-positive neurons in the subgranular zone by the length of the subgranular zone in each image (cells/mm). Only dark brown-stained cells were counted. Cell counting was blindly conducted by a technician who was unaware of the identity of each slide. 

### Collection and sectioning of cochleae and toluidine blue staining

Cochleae from ears exhibiting an intact tympanic membrane were decalcified in 10% EDTA for approximately two weeks, with fresh solution exchanges every other day. The cochleae were then embedded in paraffin and sectioned at 6 µm. Every twentieth section from each cochlea was mounted onto a slide (total of 10 slides). The distance between two adjacent sections on each slide was about 120 µm. The cochlear sections were stained with toluidine blue [64]. Images used for cell counting were collected from spiral ganglia of basal and middle turns of 3-4 midmodiolar sections from each cochlea (6 cochleae in each group). Spiral ganglion cells were identified according to established criteria [64]. The number of neurons in the spiral ganglion was counted with ImageJ software, and cell density (cells/mm^2^) was calculated and statistically analyzed as detailed below. 

### Statistical analysis

All parameters measured are expressed as means ± standard error of the mean (SEM). One-way (biomarker data) or two-way (ABR and DPOAE data) ANOVA (SPSS 14.0 for windows) was used to determine if there were statistically significant differences among the three experimental groups (NC, B, and B/T) at each time point. When a significant difference among groups was found, a post hoc test was used to determine if there were statistically significant differences between group pairings (i.e. NC vs. B; NC vs. B/T; B vs. B/T at each time point), and *p*-values were corrected for multiple comparisons. For these comparisons, the more conservative Bonferroni test was used in the ABR and DPOAE data analyses due to the smaller number of data sets, while the Tukey HSD test was applied in the biomarker and hair cell counting data analyses, where the number of statistical comparisons was more expansive. Statistical analyses were conducted using GraphPad Prism 4 software (GraphPad Software, Inc., La Jolla, CA). A *p*-value of less than 0.05 was considered to be significant.

## Results

### Antioxidant treatment reduced blast-induced ABR threshold shifts, DPOAE level shifts, and hair cell loss

The ABR, DPOAE, and hair cell counting results have been detailed in our previous report [54] and are summarized in Table 1. In general, we found that antioxidant treatment significantly reduced both ABR threshold and DPOAE level shifts, as well as reduced blast-induced hair cell loss. ABR threshold shifts in the antioxidant treatment group were about 10 dB lower at 24 hours and 20 dB lower at 7 and 21 days when compared to the untreated blast exposed group (*p* < 0.01 or < 0.001). Significant recovery in ABR threshold shifts in the antioxidant treatment group was observed at all test frequencies (2–16 kHz) at 7 and 21 days after blast exposure (*p* < 0.01 or 0.001, Table 1 and [54]).

**Table 1:** Comparison of mean ABR threshold shifts, DPOAE level shifts, and hair cell loss in the blast and blast/treatment groups (21 days after blast exposure).

	Blast	Blast/Treatment	*p* value
ABR threshold shift (2-16 kHz)	31.33 ± 1.00 dB	10.58 ± 0.70 dB	< 0.001
DPOAE level shift (2.2-15.3 kHz)	23.69 ± 1.50 dB	7.27 ± 0.77 dB	< 0.001
Outer hair cell loss (5-36 kHz)	39.68 ± 2.19 %	7.75 ± 1.12 %	< 0.001
Inner hair cell loss (5-36 kHz)	1.39 ± 0.21 %	0.17 ± 0.06 %	< 0.001

A significant decrease in DPOAE level shifts was found in the treatment group (7.5-15 dB) at 7 days after blast exposure in the higher frequency range of 4-16 kHz when compared to the blast group (20-28 dB, *p* < 0.05 or 0.01). At 21 days after blast exposure a significant decrease in the level shift was seen at all test frequencies in the treatment group (2.5-13.5 dB) compared to the blast group (15-30 dB, *p* < 0.05, 0.01 or 0.001, Table 1 and [54]).

There was also a significant difference in average outer hair cell loss observed between the antioxidant-treated and untreated groups at 7 (*p* < 0.05) and 21 (*p* < 0.001) days after blast exposure. At 21 days after blast exposure, significant reduction in outer hair cell loss in the region corresponding to 5–36 kHz was observed in the antioxidant-treated group compared to the untreated group (*p* < 0.001). Significant reduction in inner hair cell loss in the same area was also observed in the treatment group compared to the untreated group (*p* < 0.001, Table 1 and [54]). 

### Antioxidant treatment reduced production of an oxidative stress biomarker in the blast-exposed brain

**Figure 1:**
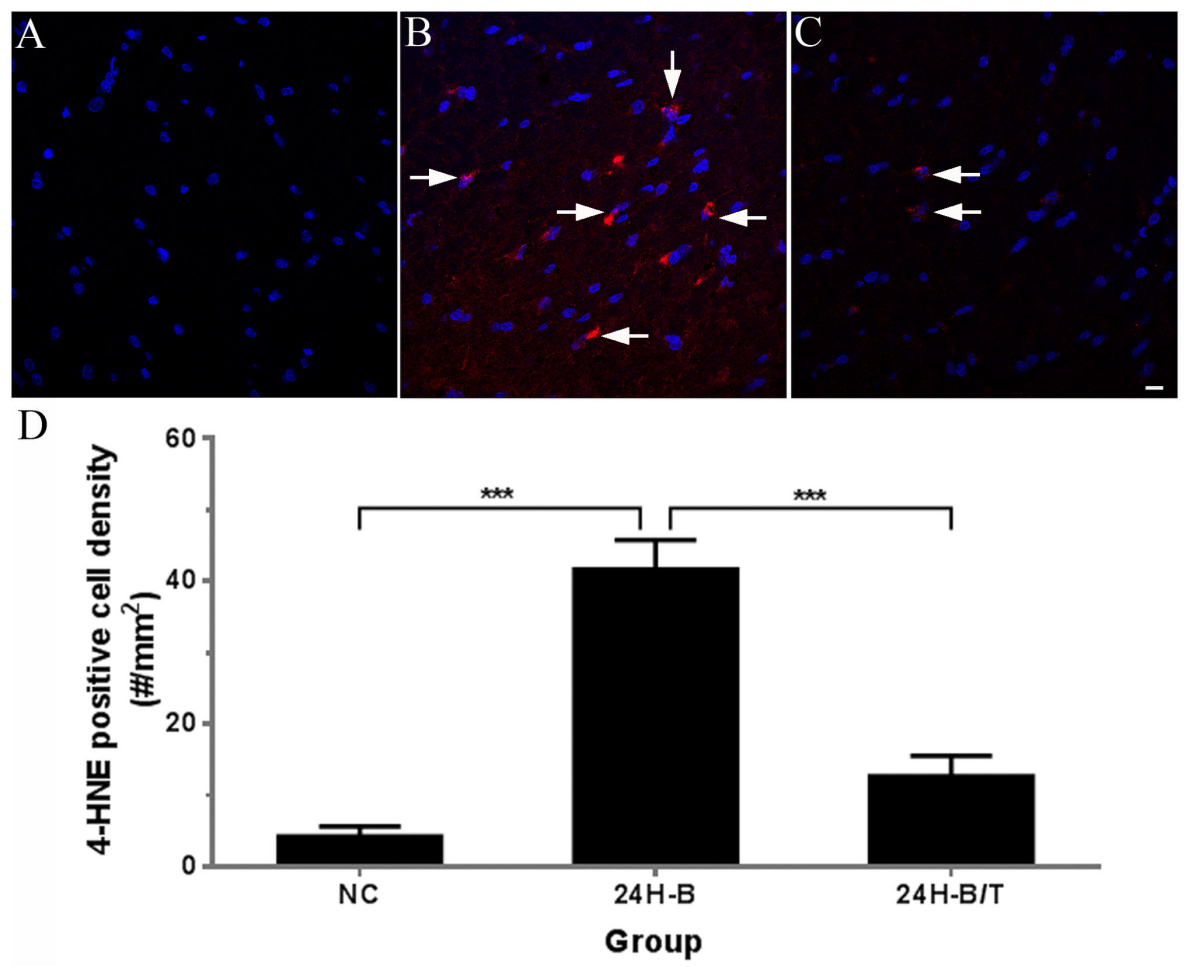
Examples of 4-HNE immunofluorescence images obtained from the dorsal hippocampal commissure of the hippocampus from either NC (A), 24H-B (B) or 24H-B/T (C) groups. No positive 4-HNE staining was observed in the NC group. Many 4-HNE positive cells were observed in the 24H-B group (arrows in B), and a reduced number of 4-HNE positive cells were observed in the 24H-B/T group (arrows in C). 4-HNE positive cells in the hippocampus were quantified and statistically analyzed (D). Four to six sections from each rat brain (6 rats in each group) were used in this analysis. Significantly increased numbers of 4-HNE-positive cells were found in the 24H-B group compared to the NC group (*p* < 0.001). Significant differences were also found between the 24H-B and 24H-B/T groups (p < 0.001), suggesting that antioxidant treatment reduced 4-HNE production in the hippocampus at this time point after blast exposure. *** indicate p < 0.001. Error bars represent standard error of the means. Scale bar in C = 10 µm for A-C.

To examine the extent of oxidative stress that is induced in brain tissue from our bTBI experimental approach, 4-HNE production was evaluated by fluorescence microscopy. Positive blast-induced 4-HNE immunolabeling was observed primarily in the hippocampus (dorsal hippocampal commissure, Figure 1B) and the forceps major corpus callosum near the lateral ventricle, with lesser staining in the cerebral cortex (data not shown), 24 hours following bTBI. 4-HNE-positive immunolabeling was also observed in these regions of the brain after only 3H following bTBI, albeit to a lesser extent than in the corresponding brain sections of the 24H groups (data not shown). In the cohort that was treated with the antioxidant combination of NAC and HPN-07 one hour after blast-exposure, the extent of 4-HNE labeling was markedly reduced at each of these sites (Figure 1C).

Fluorescence immunolabeling was conducted in hippocampal sections of all animals in the 24H post-blast (B), 24H post-blast plus treatment (B/T), and NC groups, and the resultant quantitative summaries of 4-HNE-positive cells were used for density calculations and statistical comparisons between cohorts. As summarized in Figure 1D, in comparison to the NC group (4.36 ± 1.32 cells/mm^2^), blast exposure resulted in a dramatic increase (41.79 ± 4.02) in 4-HNE positive-cells in the hippocampus 24 hours after bTBI (24H-B, *p* < 0.001). These quantitative analyses also underscore the significant reduction in 4-HNE-positive cells in the hippocampus in blast-exposed animals subsequently treated with NAC and HPN-07 (24H-B/T, 12.79 ± 2.79, *p* < 0.001, *F* (2, 77) = 43.94, Figure 1D).

### Antioxidant treatment reduced c-fos expression in brain cortex

**Figure 2:**
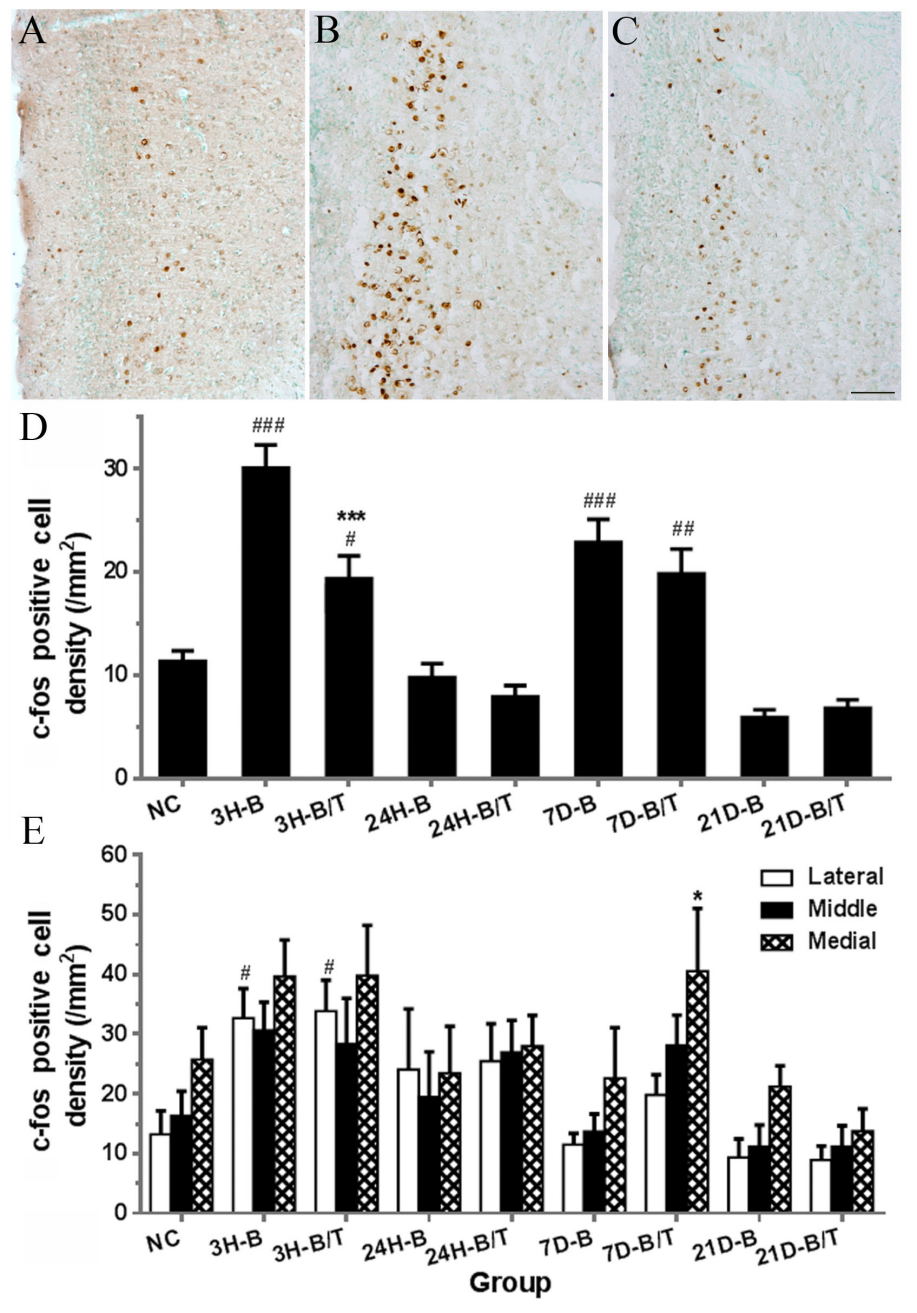
Examples of c-Fos immunostaining images obtained from the granular RC of the NC (A), 3H-B, (B), and 3H-B/T (C) groups by light microscopy. Few c-fos-positive cells were observed in layer three in the cortex of the NC group (A). Numerous c-fos-positive stained cells were seen in layer three in the cortex of the 3H-B group (B), and decreased numbers of c-fos-positive cells were seen in the cortex of the 3H-B/T group relative to the 3H-B group (C). C-fos-positive cells in the RC were quantified and statistically analyzed (D). Four to six sections (8-12 images) from each rat brain (6 rats in each group) were used in this analysis. Significantly increased numbers of c-fos-positive cells were found in the 3H-B and 7D-B groups compared to the NC group (all *p* < 0.001). Significant differences were also found between the 3H-B and 3H-B/T groups (*p* < 0.001), suggesting that antioxidant treatment suppressed c-fos upregulation in the cortex at this time point after blast exposure. However, no significant difference was found between the 7D-B and 7D-B/T groups (*p* > 0.05). C-fos-positive cells in the DCN were quantified and statistically analyzed (E). Significantly increased numbers of c-fos-positive cells were found in the lateral region of the DCN in the 3H-B and 3H-B/T groups compared to the NC group (all p < 0.05), however no antioxidant treatment effect was observed in the DCN at this time point (all p > 0.05). ### indicates p < 0.001 compared to normal controls. *** indicate p < 0.001 compared to the blast only group. Error bars represent standard error of the means. Scale bar in C = 500 µm for A-C.

C-fos is an immediate-early gene that is widely used as a marker for neuronal activity. Exposure to impulse noise has been shown to induce prolonged c-fos expression in the auditory cortex (AC) and the DCN [65], as well as in the cerebral cortex, the thalamus, and the hippocampus [66]. To determine whether our bTBI model resulted in induced c-fos expression, brain sections from normal controls and blast-exposed animals were immunostained with a c-fos antibody and subjected to comparative histological analyses. Few c-fos-positive cells were observed in the cortex (the RC and AC, Figure 2A), the DCN, the IC, and the hippocampus of normal control brains. In comparison, the degree of c-fos-positive immunostaining was strikingly higher in the RC of the brain in blast-exposed animals at three hours post-bTBI compared to normal controls (Figure 2B, and 2D, *p* < 0.05 or 0.01). We found that antioxidant treatment significantly attenuated c-fos expression in the RC in blast-exposed animals over this same time course (*p* < 0.001, *F* (8, 584) = 27.93, Figure 2C and 2D). Second peaks of RC c-fos expression were observed 7 days after blast exposure in both blast-exposed (*p* < 0.001) and blast-exposed plus antioxidant treatment (*p* < 0.01) groups when compared to the NC group (Figure 2D). However, no treatment effect was observed at this time point (*p* > 0.05). In contrast to the bTBI effects observed in the RC on c-fos expression, the observed density of c-fos-positive cells in the AC did not reflect significant induction of this immediate-early biomarker in this region of the brain 3 hours following blast-exposure (*p* > 0.05, Table S1). 

In comparison to the normal control group, c-fos expression was significantly increased in the lateral region of the DCN three hours after blast exposure (all *p* < 0.05) but not in the middle and medial regions (all *p* > 0.05, Figure 2E). However, we were unable to detect any significant changes in c-fos expression in the DCN at later time points in the blast-exposed cohort (from 24h to 21d, all *p* > 0.05). In contrast to observations in the RC at three hours post-blast, antioxidant treatment was not able to suppress the blast-induced expression of c-fos in the lateral region of the DCN (*p* > 0.05, Figure 2E). Moreover, we unexpectedly observed a greater degree of c-fos expression in the medial region of the DCN in the antioxidant-treated, blast-exposed group in comparison to the untreated blast-exposed cohort (*p* < 0.05, *F* (8,343) = 6.77, Figure 2E). No treatment effect was observed at other time points after antioxidant treatment (all *p* > 0.05, Figure 2E). 

While we observed a significant increase in c-fos expression in the hippocampus after blast exposure compared to the NC group (*p* < 0.001), no antioxidant treatment effect was observe in this region of the brain at this time point (*p* > 0.05, Table S1). We did not observe any significant changes in c-fos expression in the IC in either the B or B/T cohorts 3 hours post-blast (*p* > 0.05, Table S1). 

### Antioxidant treatment reduced GFAP expression in the cochlear nucleus

**Figure 3:**
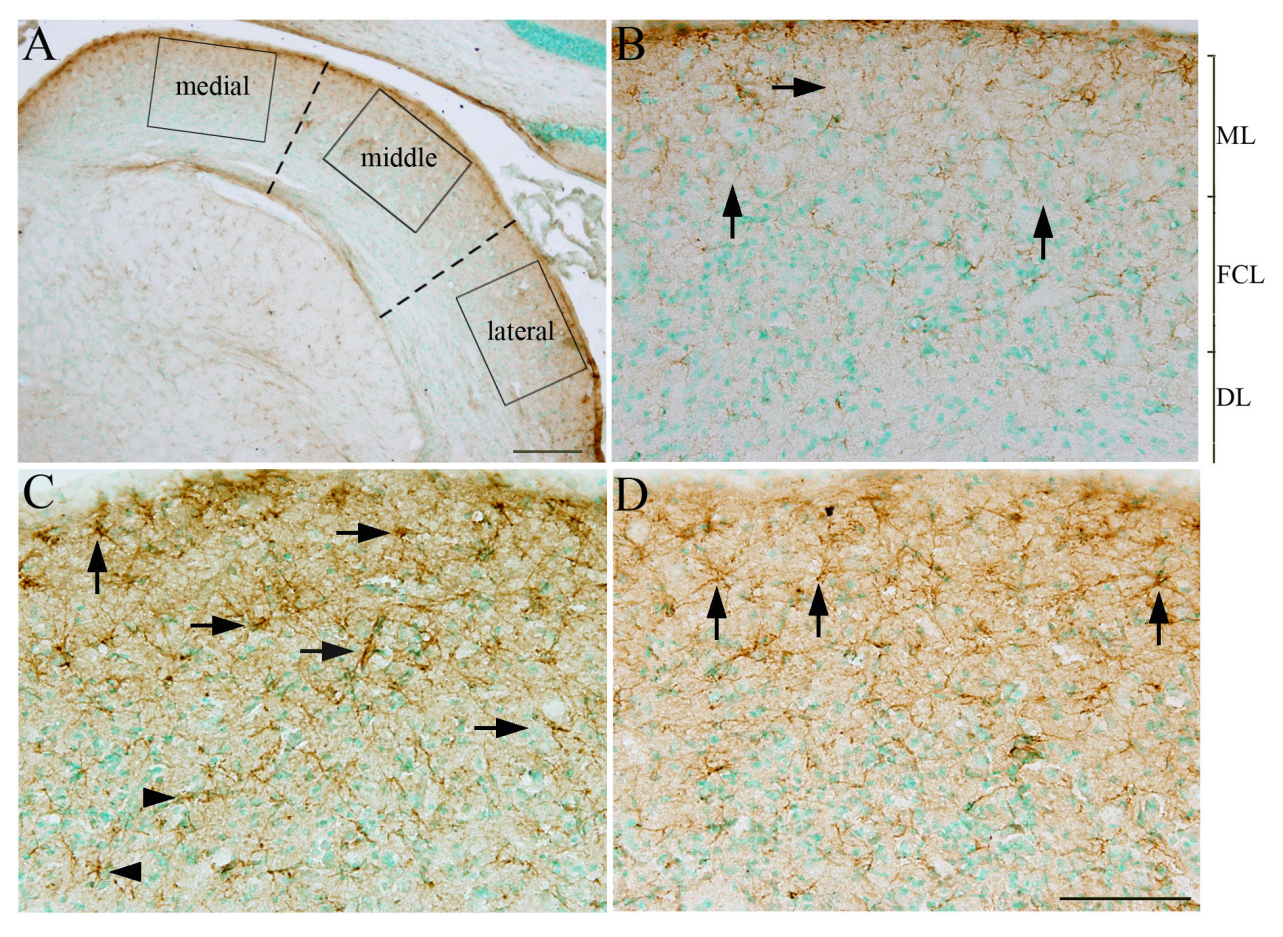
A low magnification image of the DCN is shown in A. The DCN is divided into three parts (*dashed* *lines* in A): *medial*, *middle*, and *lateral*. The *squares* in A indicate where images were collected for cell counting. Examples of GFAP staining (arrows in B-D) in the *middle* region of the DCN of the NC (B), 21D-B (C) and 21D-B/T (D) groups. ML, FCL, and DL in B demarcate the molecular layer, fusiform cell layer, and deep layer, respectively. Scale bar in A = 500 µm, in D = 200 µm in for B-D.

**Figure 4:**
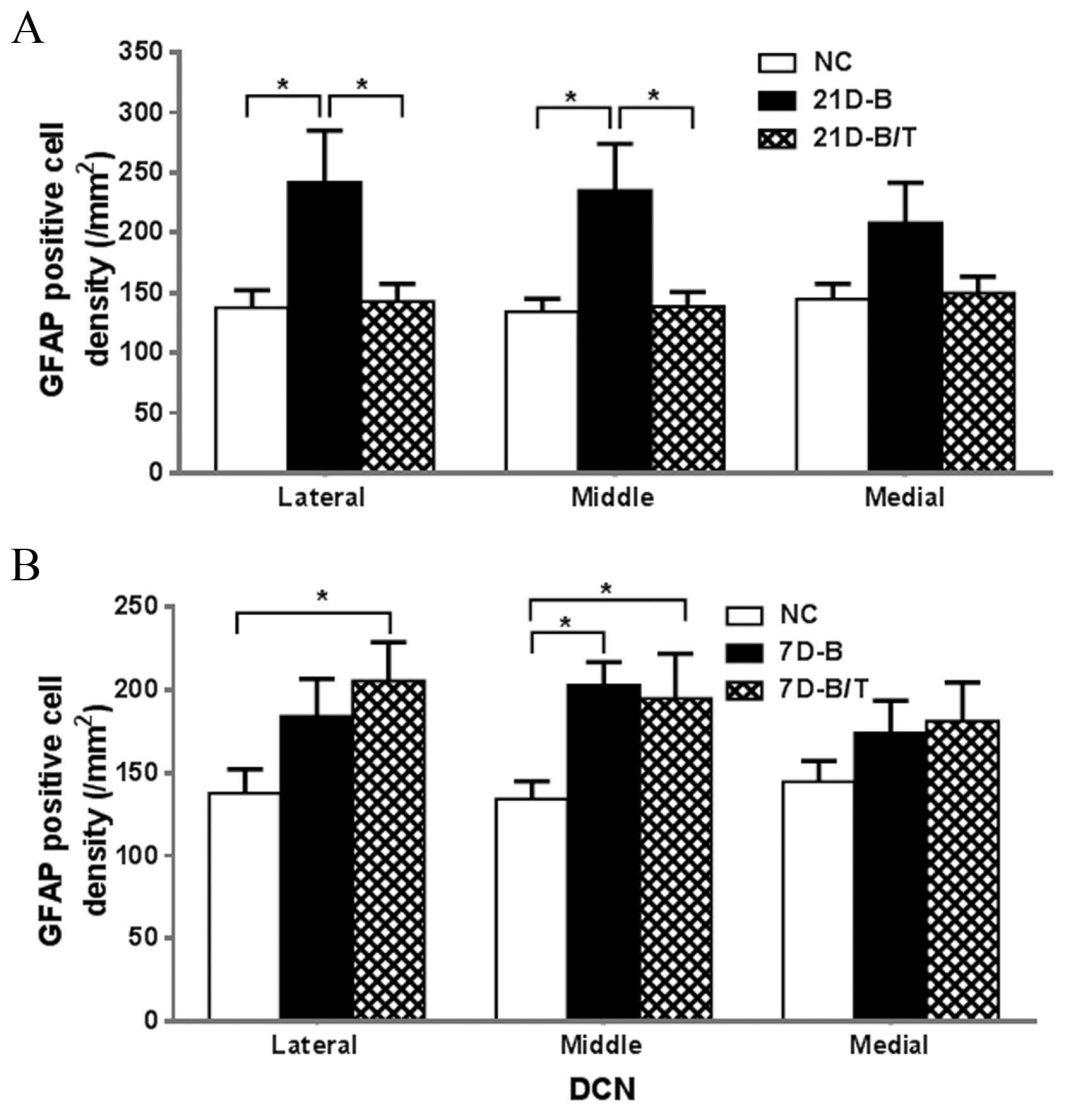
GFAP-positive cells in the DCN of the NC, 21D-B, and 21D-B/T (A) or 7D-B, and 7D-B/T (B) groups were quantified and statistically analyzed. Two to three DCN sections from each rat brainstem (6-7 rats in each group) were used in these analyses. Significantly increased GFAP expression is observed in the *lateral* and *middle* regions of the 21D-B and 7D-B groups compared to the NC group (all *p* < 0.05). Decreased numbers of GFAP-positive cells were observed in these two regions in the 21D-B/T group compared to the 21D-B group (A, all *p* < 0.05), suggesting a treatment effect in these regions at this time point after blast exposure. However, no treatment effect was observed in GFAP expression in these regions 7 days after blast exposure (B, all *p* > 0.05). No significant difference was observed in the medial region of the DCN of the 21D or 7D groups compared to the NC group (all *p* > 0.05). Error bars represent standard error of the means. * indicate *p* < 0.05.

GFAP has been considered to be a marker of active astrocytes and indicates a repair-regenerative process after neuronal damage [23]. Active astrocyte gliosis appears to be a prominent early stage feature of blast-induced brain damage. As such, we examined GFAP expression in the brains of rats subjected to our model of blast-induced trauma. While a modest GFAP staining was observed in the DCN of normal controls (arrows in Figure 3B), most of these positively-stained cells were located in the superficial layers (molecular and fusiform cell layers), with only a few positively stained cells located within the deep layer (Figure 3B). Significantly increased GFAP expression, with penetrance into the deep layer, was observed in the lateral and middle regions of the DCN 21 days after blast exposure as compared to the NC group (all *p* < 0.05, Figures 3C and 4A). No significant change in GFAP expression was observed at this time point in the medial region after blast exposure (*p* > 0.05, Figure 4A). Antioxidant treatment significantly attenuated the induced GFAP expression in the middle and lateral regions of the DCN 21 days after blast exposure (all *p* < 0.05, *F* (2, 132) = 12.70, Figures 3D and 4A). Significant increases in GFAP expression in the DCN were also observed in the middle and lateral regions examined at an earlier time point (7 days) after blast exposure (all *p* < 0.05, *F* (2, 113) = 8.50). However, in contrast to observations made at 21 days post-blast-exposure, no treatment effect was observed in these regions of the DCN at this earlier time point (all *p* > 0.05). Consistent with later time points, there was no significant change in GFAP expression in the medial region of the DCN 7 days after blast exposure (all *p* > 0.05, Figure 4B). Taken together, these results suggest that antioxidant treatment exhibits a delayed therapeutic effect on blast-induced GFAP expression in the DCN after a 14 psi blast exposure. 

Svetlov and coworkers previously documented induction of regionally-specific GFAP expression in the hippocampus that peaked at 7 days and persisted up to 30 days after a high (52 psi) blast exposure. However, no increased GFAP expression was observed in the cortex (i.e. the AC) in their study [23]. In the present study, we also observed significantly higher GFAP expression levels in the hippocampus following blast exposure (all *p* < 0.01, Table S2). However, no apparent antioxidant treatment effect was observed on the increased hippocampal GFAP levels in blast-exposed animals (*p* > 0.05, Table S2). Similar to the observations made by Svetlov and colleagues, we observed no significant difference in GFAP levels in the AC between the experimental cohorts (*p* > 0.05, Table S2). Similarly, we observed no significant differences in GFAP levels in either the central nucleus of the IC or in the RC in rats subjected to bTBI (all *p* > 0.05, Table S2). These results indicate that GFAP expression was not significantly altered in the AC, IC, or RC 21 days after blast exposure or after blast exposure plus antioxidant treatment. 

### No neuron loss in the DCN after blast exposure

Increased GFAP expression in the DCN after blast exposure may be indicative of neuronal injury [67,68]. To assess potential loss of neurons in the DCN, relevant sections in each experimental cohort were immunostained with an antibody against the neuron specific nuclear protein, NeuN. From this analysis, we were unable to discern any significant difference in NeuN-positive neuronal densities between the experimental groups within any region of the DCN (all *p > 0.05*, Table S3). These results indicate that there was no apparent neuron loss in the DCN 21 days after blast exposure or following blast exposure plus antioxidant treatment, suggesting that there was reactive astrocytosis without accompanying neuron loss in the DCN after blast exposure. 

### Antioxidant treatment reduced axonal injury in the brain

**Figure 5:**
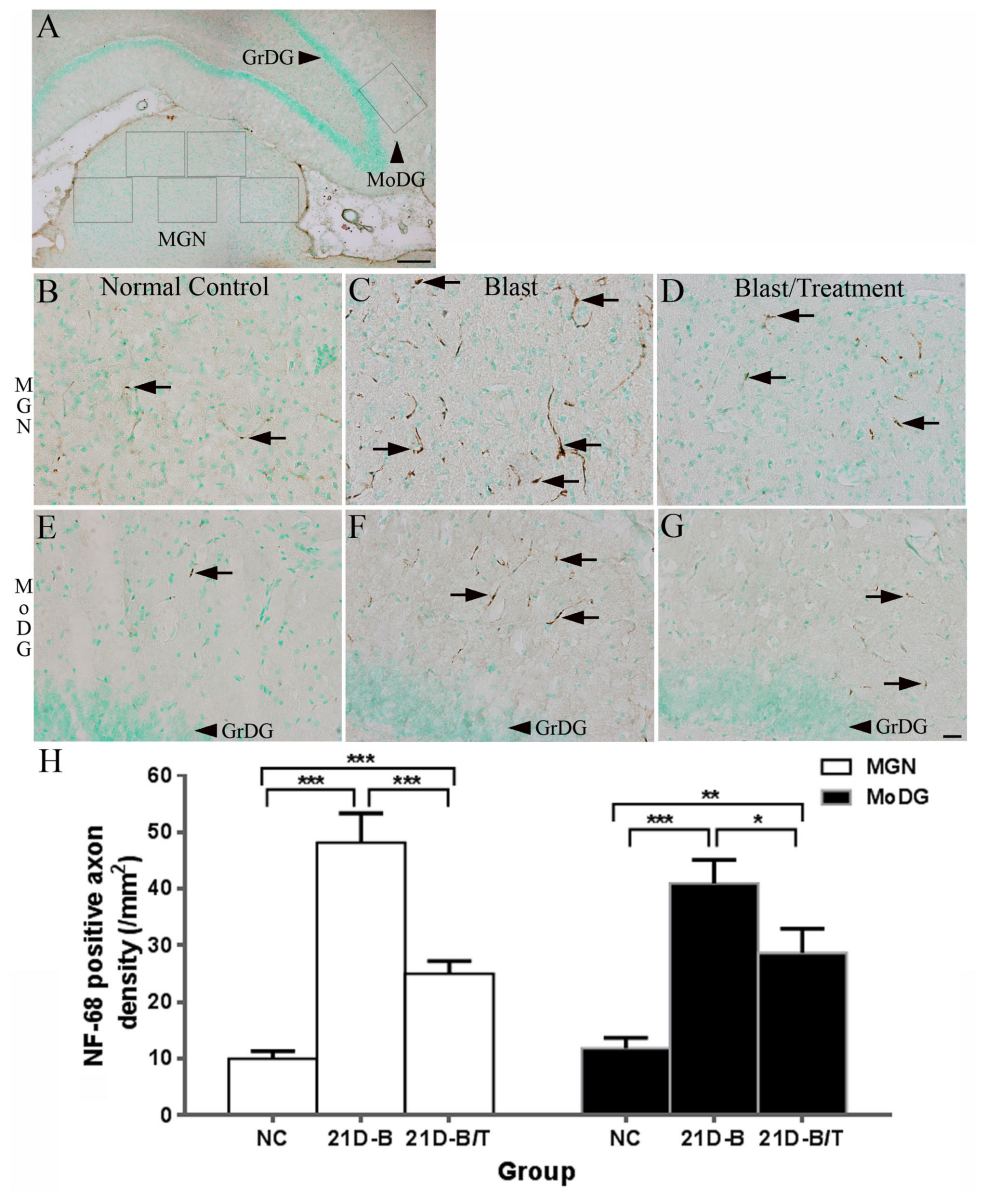
Examples of NF-68 expression (arrows in B-G) in the MGN (B-D) and MoDG of the hippocampus (E-G) from the NC (B and E), 21D-B (C and F) and 21D-B/T (D and G) cohorts. A low magnification image of the relevant brain region for NF-68 staining is shown in A. The *squares* in A indicate where images were collected from the MoDG and the MGN for NF-68-positive axon counting and statistical analyses (H). Four to five images were taken from each MGN section and 3-4 MGN sections from each rat brain were used. One image was taken from each MoDG section and six MoDG sections from each rat brain (6 rats in each group) were used in these analyses. Significantly increased numbers of NF-68-positive axons were observed in the MGN and MoDG of the 21D-B group compared to the NC group (all *p* < 0.001). Significantly decreased numbers of NF-68-positive axons were observed in the MGN and MoDG in the 21D-B/T group relative to the 21D-B group (*p* < 0.001 or 0.05). Error bars represent standard error of the means. Scale bar = 50 µm in G for B-G, = 500 µm in A.

To probe for axonal injury following blast-exposure, we immunostained brain tissue for NF-68 and APP. In normal rat brains, weak NF-68 positive staining was observed in the MGN and MoDG (arrows in Figure 5B and 5E). A significantly increased number of NF-68-positive axons was observed in both of these regions 21 days after blast exposure compared to normal controls (all *p* < 0.001, Figure 5C, 5F, and 5H). Axons in the MGN and MoDG of blast-exposed animals exhibited multi-beaded degeneration (arrows in Figure 5C and F) [58]. Antioxidant treatment significantly reduced the number of NF-68-positive axons in both the MGN (*p* < 0.001, *F* (2, 345) = 43.08) and MoDG (*p* < 0.05, *F* (2, 104) = 16.39) at 21 days after blast exposure (Figure 5D, 5G, and 5H). There was no significant increase in NF-68 expression in the MGN and MoDG at earlier time points after blast exposure (3H, 24H, and 7D) nor were there any significant changes in NF-68 expression in the IC, DCN, or ventral cochlear nucleus at any time point after blast exposure compared to normal controls (data not shown). 

**Figure 6:**
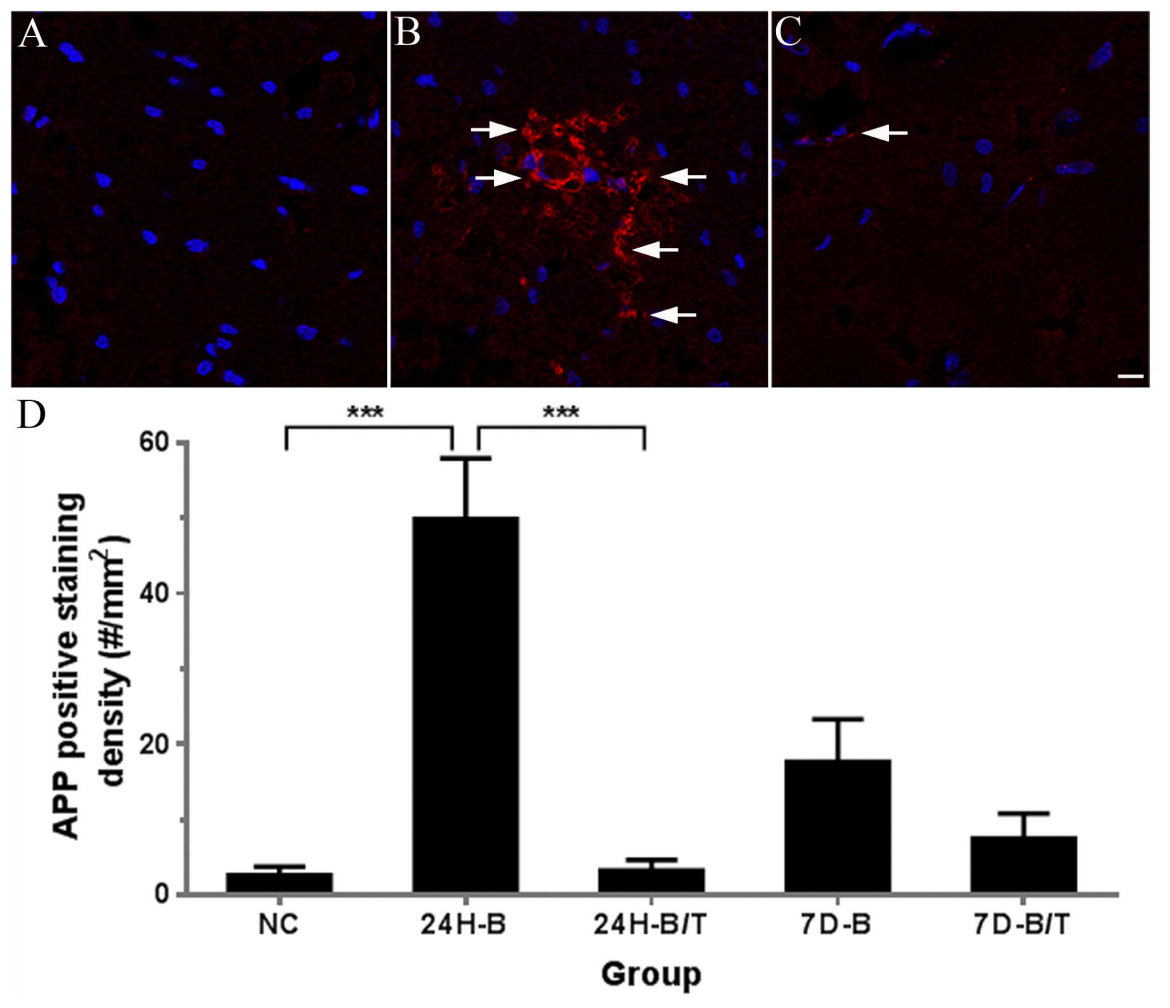
Examples of APP immunolabeling in the hippocampus of the NC (A), 24H-B (B) and 24H-B/T (C) groups. No positive APP staining was observed in the hippocampus of normal controls (A). Strong positive APP labeling was observed in the hippocampus of the 24H-B group (arrows in B). Decreased APP expression was observed in the hippocampus of the 24H-B/T group relative to the 24H-B group (arrows in C). APP-positive labeling in the hippocampus was quantified and statistically analyzed (D). Two to three hippocampal sections from each rat brain (6 rats in each group) were used in these analyses. Significantly increased APP expression was observed in the hippocampus of the 24H-B group compared to the NC group (*p* < 0.001). An antioxidant treatment effect was found at 24 hours after blast exposure (*p* < 0.001), however no significant difference was observed between the treated and untreated groups 7 days after blast exposure (7D-B v.s. 7D-B/T, all *p* > 0.05). Error bars represent standard error of the means. Scale bar = 10 µm in C for A-C. *** indicate *p* < 0.001.

Previous studies have demonstrated a correlation between bTBI and increased APP expression, consistent with blast-induced axonal injury [20,32,69]. While we observed very low levels of positive APP staining in normal brain tissues (Figure 6A), animals in the blast-exposed cohort exhibited marked induction of hippocampal APP expression as early as 24 hour post-trauma (Figure 6B and 6D, *p* < 0.001). Remarkably, antioxidant treatment seemingly blocked the blast-induced upregulation of APP production in the hippocampus over this same time period (*p* < 0.001, *F* (4, 25) =18.19, Figure 6C and 6D). Moreover, combinatorial antioxidant treatment also blocked increases in bTBI-related APP upregulation in the cortex, including the AC, that were observed during this post-exposure interval (data not shown). It is worth noting that these blast-induced changes in APP expression were seemingly transient, as no significant increases in APP expression in either of these tissues were evident at later time points (i.e. 7 - 21days after blast exposure, *p* > 0.05, Figure 6D). 

### Neurogenesis was not impaired by blast exposure

In order to assess whether bTBI impacted neurogenesis in our model system, we examined the relative levels of the neuronal migration biomarker, doublecortin, in blast-exposed brain tissues. From this analysis, we found that doublecortin-positive staining was primarily concentrated in the subgranular zone of the hippocampus and within the deep layer and fusiform cellular components of the DCN in normal controls. Comparative analyses with blast-exposed animals revealed that there was no significant difference in doublecortin levels between any of the three experimental cohorts (all *p* > 0.05, Table S4), thus indicating that neurogenesis was not significantly impaired in our model of bTBI. 

### No positive caspase 3 staining in the brain after blast exposure

Evidence for blast-induced apoptotic neuronal death has previously been reported for deep brain regions within a few hours or days after 117, 153 or 515 kPa (^≈^ 16, 22.2 or 75 psi, respectively) blast exposures [20,42]. Apoptotic neuronal death has only been documented in the brain after high-overpressure blast exposures (16, 22.2 or 1450 psi) [42,45]. Low level blast exposure (7.1 or 11.3 psi) has been shown to result in non-apoptotic (caspase 3-negative) DNA damage (TUNEL positive) in oligodendrocytes and astrocytes in the brain [43]. In the present study, no positive caspase 3 staining was observed in either the brain or brainstem at any time point after blast exposure or blast exposure plus treatment. This result suggests that apoptotic cell death is not involved in the brain damage induced by the blast overpressure produced in our experimental system.

### No spiral ganglion neuron loss 21 days after blast exposure

Comparative analyses with blast-exposed animals revealed that there was no significant difference in spiral ganglion cell densities in basal and middle turns between any of the three experimental cohorts (all *p* > 0.05, Table S5). Thus, while we observed significant impairment to auditory function in our model of bTBI, there was no apparent loss of spiral ganglia for up to 21 days under these conditions. These results suggest that changes in the sensorineural functionality of blast-exposed cochleae are not directly attributable to loss of neurons within the associated spiral ganglia.

## Discussion

### Blast brain damage examined by biomarkers

In this preliminary study, blast exposure induced upregulation of several stress-related biomarkers in the brain and brainstem of rats. These biomarkers included 4-HNE, c-fos, GFAP, APP and NF-68. 4-HNE is a lipid peroxidation marker. Our results and previous studies seem to indicate that oxidative stress is rapidly elevated in the brain after blast exposure (Figure 1 and [22,28,36,38]). Significant increases in 4-HNE levels in rat brains were previously documented 3-24 hours after 120 or 123 KPa (17.40 or 17.84 psi) blast exposures [22,36]. Increased 4-HNE levels have also been observed in the organ of Corti after acute acoustic trauma [70,71]. These observed increases in oxidative stress may cause mitochondrial injury, activation of cell death pathways and mediators of inflammation, glutamate excitotoxicity, and increased levels of lipid peroxidase [10,50,72-77]. Oxidative stress can affect the injured brain by acting through the brain-derived neurotrophic factor (BDNF) system to affect synaptic plasticity and cognition [78]. Oxidative stress may also play a key role in the breakdown of the blood-brain barrier induced by blast exposure [22,36].

C-fos is an immediate early gene and biomarker for neural activity. Noise-induced c-fos expression in the central auditory system has been shown previously to be noise intensity-dependent and demonstrates tonotopic organization in some nuclei (i.e. the DCN, the ventral cochlear nucleus, and the medial nucleus of the trapezoid body) [79-82]. Exposure to impulse noise has been shown to lead to prolonged c-fos expression in the cerebral cortex, the thalamus, the hippocampus, and the DCN [63,64]. In the present study, broad upregulation of c-fos was observed in the brain after blast exposure (Figure 2). Increased numbers of c-fos-positive cells were observed in the RC, the hippocampus, the cochlear nucleus, and IC immediately after blast exposure. In the RC, a second peak of apparent c-fos upregulation was observed at 7 days after blast exposure (Figure 2D). We have previously observed a similar secondary peak of delayed c-fos expression in the ventral cochlear nucleus 24 hours after intense noise exposure (our unpublished data). These observations may reflect long-term changes in neural processing pathways, such as changes in inhibitory interneurons [83,84]. The RC is involved in spatial learning and navigation. Disrupted spatial navigation has previously been documented in rats 2-3 days following blast exposure [85,86] and can persist for months to years after blast exposure in humans [87].

Elevated GFAP levels have been documented in the hippocampus and cerebral cortex (i.e. the prefrontal and primary motor cortex) of brains, as well as in serum, after blast exposure [16,23,41,42,88]. The GFAP level in serum may be a good biomarker to predict outcome after brain injury [23]. Significantly increased numbers of GFAP-positive astroglial cells could be detected adjacent to a cortical contusion from 1 day up to 4 weeks after human brain injury [89], and thus, the quantity of astrocytes, indentified by GFAP immnuostaining, might be closely related to the level of blast exposure [42] and the severity of posttraumatic brain injury [90]. In the present study, increased expression of GFAP was observed in the hippocampus (CA2 region), the AC, the DCN, and the IC after blast exposure (Figures 3 and 4 and data not shown). Increased GFAP expression in the DCN following blast exposure could be indicative of neuronal injury [67,68]. However, upon quantifying neurons in the DCN of blast-exposed animals, using a NeuN antibody, we observed no neuron loss at 21 days after blast exposure, at a time point when GFAP expression in the DCN significantly increased (Figures 3C and 4A). This result may indicate astrocytosis in the DCN without corresponding neuron loss after blast exposure. However, while anti-NeuN antibody stains a majority of neurons in the CNS [91]; the present results cannot formally rule out some subtype of neuron loss in the DCN after blast injury.

Positive or increased expression of axonal injury biomarkers (APP and NF-68) were also observed in the hippocampus, the AC, and the MGN (Figures 5 and 6). The hippocampus belongs to the limbic system and plays important roles in the consolidation of information from short-term memory to long-term memory and spatial navigation. The MGN is part of the auditory thalamus and represents the thalamic relay between the IC and the AC. It is thought that the MGN also influences the direction and maintenance of attention. Axonal and neural injury in these regions may be involved in memory loss and disorientation observed after blast exposure [14,85-87,92]. These results indicate that the central auditory pathway is also vulnerable to blast exposure. Therefore, injuries in the central auditory pathway may also be involved in sensorineural hearing loss detected after blast exposure [54].

Results of the biomarker study presented herein indicate a regional specificity in neuronal and axonal injury in the brain after blast exposure. This regionally-specific biomarker expression pattern has been reported in previous studies [25,88,93]. Regional brain hypometabolism has been documented in Iraq War veterans with repeated episodes of mild TBI from explosive blasts, which may explain the chronic post-concussive symptoms documented in many of these soldiers [94]. Results of GFAP expression in the DCN suggest that the lateral and middle regions of the DCN may be more sensitive to blast exposure than the medial region (Figure 4). Our laboratory previously documented a similar regional specificity with respect to synaptic degeneration in the DCN after noise exposure [62]. Therefore, biomarker studies may provide useful information for understanding the mechanisms of bTBI and developing treatment methodologies.

Our results suggest that neurogenesis in the hippocampus and in the DCN was not impaired by the blast exposure model (14 psi) used in the present study at the time point examined (21 days after blast exposure). A previous study provided evidence that neurogenesis genes are downregulated in the hippocampus 24 hours after a 130 - 260 KPa (18.85 - 37.70 psi) blast exposure [47]. However, increased levels of doublecortin have been detected in the hippocampus as late as two months post-blast injury [59]. Spiral ganglion cell loss has also been reported at a sampling interval five weeks after blast exposure (172 dB, [95]). The intensity of blast exposure used in the present study was 14 psi (196 dB), and neurogenesis and spiral ganglion neurons were examined 21 days after blast exposure. Thus, a longer term study may be needed to discern whether our model of blast exposure and antioxidant treatment induce delayed response patterns on neurogenesis in the hippocampus and spiral neuron loss in the spiral ganglion. 

### Effects of antioxidants on brain biomarker expression after blast exposure

Some preventive or treatment measures against blast-induced brain damage have been reported. Aminoguanidine, an inducible nitric oxide synthase inhibitor and neuroprotective agent, facilitated the recovery of neuro-behavioral changes (coordination and grip strength) induced by blast exposure in rats and reduced the number of degenerated cortical neurons [12]. The nonselective caspase inhibitor N-benzyloxycarbonyl-Val-Ala-Asp-fluoromethylketone (Z-VAD-FMK) has been shown to prevent apoptotic neuron death induced by high-overpressure shock waves (>10 MPa) in the rat brain [45]. Minocycline (an anti-inflammatory drug) treatment has shown efficacy in normalizing serum and tissue levels of many biomarkers, including GFAP, and may prevent the development of neuro-behavioral abnormalities [16]. Additionally, low-pressure hyperbaric oxygen therapy significantly improves atypical neural symptoms, abnormal physical exam findings, cognitive testing, and quality-of-life measurements for blast-induced post-concussion syndrome and post-traumatic stress disorder [96]. 

We chose a combination of two antioxidants, NAC and HPN-07, for this study. NAC functions to increase the intracellular pool of the antioxidant glutathione [75]. HPN-07 is a free radical spin-trapping agent that has exhibited efficacy as a neuroprotectant and inhibits upregulation of inducible nitric oxide synthase, decreases glutamate excitotoxicity, and may decrease cell death [97]. Results of the present study indicate that antioxidant treatment significantly reduced oxidative stress in the brain, as evidenced by marked decreases in blast-induced lipid peroxidation (i.e. 4-HNE levels, Figure 1). This treatment effect on 4-HNE production was also observed previously in the organ of Corti following noise exposure [70,71]. By reducing oxidative stress, antioxidant treatment may, therefore, reduce mitochondrial injury, activation of cell death pathways, and mediators of inflammation and glutamate excitotoxicity to provide protection to the brain and inner ear. 

In the present study, regionally-specific treatment effects were observed in the CNS. Treatment effects were observed in the RC, the AC, the hippocampus, the MGN and the DCN. The antioxidants reduced the expression levels of the immediate early gene, c-fos, in the RC; blast-induced GFAP levels in the DCN; and axonal injury in the hippocampus, the AC, and the MGN. The results reported herein suggest that this antioxidant treatment regimen may not only provide protection to the inner ear [54] but also to the CNS. The hippocampus and RC are involved in spatial learning and navigation. Blast-injured animals have been shown to exhibit persistent spatial memory impairment [44,59]. Therefore, antioxidants may have the potential to treat brain injury, and thus neuropsychiatric sequelae, induced by blast exposure, such as memory loss and disorientation. However, we observed no treatment effect on c-fos expression in the RC at 7 days after blast exposure, suggesting that a longer time-course of antioxidant treatment may be needed. 

NAC is not only an antioxidant but also has anti-inflammatory and anti-apoptotic effects and restores mitochondrial functions induced by TBI [40,98-102]. Neuroprotection by NAC has been observed in animal models [103,104] and in humans [15]. A double blind, placebo-controlled clinical study has demonstrated that NAC exhibits beneficial effects on the severity and resolution of sequelae of mild bTBI [15]. NAC also attenuates ischemia/reperfusion brain injury and improves cerebral oxygen delivery and perfusion in animal models [105-108]. However, several studies have indicated that therapeutic strategies in which NAC is combined with other complementary treatments yield more robust results. For instance, co-administration of NAC with minocycline synergistically improved spatial learning, lowered interleukin-1 levels, and preserved white matter following TBI in rats [109]. NAC with nutritional supplements (i.e. sodium selenite) offered significant protection against mercury-induced oxidative stress in rats [110]. A combinatorial treatment regimen of hypothermia plus NAC has been shown to attenuate hypoxic ischemic brain injury [111]. Co-administration of NAC with 4-hydroxy phenyl N-tert-butyl nitrone (4-OHPBN, a structural ortholog of HPN-07) attenuated oxidative stress in the cochlea [70] and synaptic degeneration in the DCN associated with acute acoustic trauma [62]. Our unpublished data also suggest that a combination of NAC with HPN-07 provides synergistic protection to the peripheral auditory system.

A neuroprotective role for HPN-07 has also been observed in other animal models. HPN-07 was found to reduce loss of injured brain tissue and improved cognitive function when administered to rats after percussion-induced traumatic brain injury through inhibition of reactive oxygen species [73]. HPN-07 treatment reduced infarct volume in rat models of stroke [112-114] and lessened functional disability in a primate model of stroke [115]. HPN-07 has also been shown to reduce ischemic brain damage through suppressing apoptotic cell death pathway [112]. 

A question raised here is why the treatment effects identified by biomarker expression were only observed in some, but not all, brain regions. Multiple factors, including blast wave physics, primary and secondary brain injuries, as well as systemic pathophysiological responses to blast waves, are involved in the mechanisms of bTBI [3,25,116]. Furthermore, as a consequence of the initial mechanical impact to the brain, cerebral metabolism, blood flow, and ion homeostasis are altered. High levels of glutamate, calcium and lactate are thus released, and many cytokines are generated as a result [11,117-119]. Blast waves can cause more injury to the surface of the brain than to the deeper regions, although the hippocampus is affected as well [33]. Consistent with our results with NF-68 and APP immunostaining, silver staining, indicative of neuronal and axonal degeneration, has been shown to be prominent in some deep regions of the brain but not in the cerebral cortex under similar conditions [23]. Therefore, different mechanisms may be involved in the specific types of injuries that occur in different brain regions [21]. Different stimuli can induce c-fos expression in the brain, and the same stimuli can induce different c-fos expression patterns in different brain regions [120,121]. In the present study, the antioxidant treatment effect on c-fos expression was only observed in the RC, while the treatment effect on GFAP expression was only seen in the DCN. These results suggest different mechanisms may be involved in the expression of these biomarkers in different brain regions. The combination of NAC and HPN-07 primarily targets oxidative stress, which is one of the underlying mechanisms of bTBI. Thus, combinations of drugs that simultaneously target multiple stress pathways may elicit an even greater therapeutic response.

## Conclusion

Antioxidant treatment can provide both functional and phenoptypic protection to the peripheral auditory end organ, the cochlea [54]. Our preliminary study described herein suggests that the same antioxidant treatment may also provide a degree of protection to the central auditory pathway (the DCN and the MGN) and non-central auditory regions (the hippocampus and RC). Thus, antioxidants have the potential to treat brain injury and, thus, neuropsychiatric sequelae induced by blast exposure, such as memory loss and disorientation, under therapeutic conditions that also prevent pervasive sensorineural damage to the auditory system. Complementary performance evaluations, such as memory tests and spatial navigation, should be conducted in the future to determine whether this treatment strategy can provide functional protection to brain injuries induced by blast exposure.

## Supporting Information

Table S1 **Comparison of c-fos-positive cell densities (cells/mm^2^) in the AC, hippocampus, and IC 3 hours after blast exposure.**
(DOC)

Table S2 **Comparison of GFAP-positive cell densities (cells/mm^2^) in the AC, hippocampus, and IC 21 days after blast exposure.**
(DOC)

Table S3 **Comparison of NeuN-positive neuron densities (cells/mm^2^) in the DCN 21 days after blast exposure.**
(DOC)

Table S4 **Comparison of doublecortin-positive cell density in the hippocampus (cells/mm) or in the DCN (cells/mm^2^) 21 days after blast exposure.**
(DOC)

Table S5 **Comparison of spiral ganglion cell density (cells/mm^2^) 21 days after blast exposure.**
(DOC)
