# Supplementary material for: Effects of Antioxidant Treatment on Blast-Induced Brain Injury
Source: PLoS One. 2013 Nov 5;8(11):e80138. doi: 10.1371/journal.pone.0080138 (PMC3818243; doi:10.1371/journal.pone.0080138)
Supplement: Table S1 — Comparison of c-fos-positive cell densities (cells/mm2) in the AC, hippocampus, and IC 3 hours after blast exposure. (DOC) [file pone.0080138.s001.doc]

Supplemental Table 1. Comparison of c-fos-positive cell densities (cells/mm^2^) in the AC, hippocampus, and IC 3 hours after blast exposure.

| Brain region or nucleus | NC | B | B/T | *F* value | *p* value |
| --- | --- | --- | --- | --- | --- |
| AC | 9.24 ± 0.38 | 8.23 ± 0.32 | 6.97 ± 0.28 | (2, 56) = 0.53 | > 0.05 |
| Hippocampus | 0.94 ± 0.03 | 31.85 ± 1.17 | 38.40 ± 1.47 | (2, 77) = 12.05 | < 0.001 (NC vs. B or B/T); > 0.05 (B vs. B/T) |
| IC | 4.50 ± 0.25 | 2.32 ± 0.13 | 4.33 ± 0.35 | (2, 53) = 1.04 | > 0.05 |
