# Supplementary material for: Effects of Antioxidant Treatment on Blast-Induced Brain Injury
Source: PLoS One. 2013 Nov 5;8(11):e80138. doi: 10.1371/journal.pone.0080138 (PMC3818243; doi:10.1371/journal.pone.0080138)
Supplement: Table S2 — Comparison of GFAP-positive cell densities (cells/mm2) in the AC, hippocampus, and IC 21 days after blast exposure. (DOC) [file pone.0080138.s002.doc]

Supplemental Table 2. Comparison of GFAP-positive cell densities (cells/mm^2^) in the AC, hippocampus, and IC 21 days after blast exposure.

| Brain region or nucleus | NC | B | B/T | *F* value | *p* value |
| --- | --- | --- | --- | --- | --- |
| AC | 147.56 ± 14.78 | 180.84 ± 18.82 | 195.80 ± 18.91 | (2, 143) = 1.72 | > 0.05 |
| Hippocampus | 155.96 ±16.03 | 275.60 ± 26.11 | 222.53 ± 25.86 | (2, 91) = 6.47 | < 0.01 (NC vs. B or B/T ); > 0.05 (B vs. B/T) |
| IC | 52.70 ± 5.43 | 47.46 ± 5.00 | 57.70 ± 6.73 | (2, 63) = 0.79 | > 0.05 |
