# Supplementary material for: Effects of Antioxidant Treatment on Blast-Induced Brain Injury
Source: PLoS One. 2013 Nov 5;8(11):e80138. doi: 10.1371/journal.pone.0080138 (PMC3818243; doi:10.1371/journal.pone.0080138)
Supplement: Table S3 — Comparison of NeuN-positive neuron densities (cells/mm2) in the DCN 21 days after blast exposure. (DOC) [file pone.0080138.s003.doc]

Supplemental Table 3. Comparison of NeuN positive neuron densities (cells/mm^2^) in the DCN 21 days after blast exposure.

| DCN region | NC | B | B/T | *F* value | *p* value |
| --- | --- | --- | --- | --- | --- |
| Lateral | 344.06 ± 27.68 | 346.20 ± 25.34 | 343.75 ± 18.30 | (2, 31) = 0.81 | > 0.05 |
| Middle | 403.91 ± 25.44 | 389.04 ± 25.16 | 394.72 ± 31.54 | (2, 31) = 0.46 | > 0.05 |
| Medial | 381.52 ± 46.13 | 379.18 ± 46.30 | 365.98 ± 40.60 | (2, 31) = 0.69 | > 0.05 |
