# Supplementary material for: Effects of Antioxidant Treatment on Blast-Induced Brain Injury
Source: PLoS One. 2013 Nov 5;8(11):e80138. doi: 10.1371/journal.pone.0080138 (PMC3818243; doi:10.1371/journal.pone.0080138)
Supplement: Table S4 — Comparison of doublecortin-positive cell density in the hippocampus (cells/mm) or in the DCN (cells/mm2) 21 days after blast exposure. (DOC) [file pone.0080138.s004.doc]

Supplemental Table 4. Comparison of doublecortin-positive cell density in the hippocampus (cells/mm) or in the DCN (cells/mm^2^) 21 days after blast exposure.

| Brain region | NC | B | B/T | *F* value | *p* value |
| --- | --- | --- | --- | --- | --- |
| Hippocampus | 9.59 ± 0.4 | 7.96 ± 0.29 | 8.41 ± 0.39 | (2, 170) = 0.79 | > 0.05 |
| DCN (lateral) | 329.21± 48.16 | 359.93 ± 40.09 | 446.69 ± 56.77 | (2, 35) = 1.49 | > 0.05 |
| DCN (middle) | 517.97 ± 95.51 | 613.11 ± 76.3 | 643.47 ± 82.20 | (2, 35) = 0.60 | > 0.05 |
| DCN (medial) | 526.53 ± 115.73 | 492.66 ± 73.68 | 565.40 ± 71.52 | (2, 35) = 0.15 | > 0.05 |
