# Supplementary material for: Effects of Antioxidant Treatment on Blast-Induced Brain Injury
Source: PLoS One. 2013 Nov 5;8(11):e80138. doi: 10.1371/journal.pone.0080138 (PMC3818243; doi:10.1371/journal.pone.0080138)
Supplement: Table S5 — Comparison of spiral ganglion cell density (cells/mm2) 21 days after blast exposure. (DOC) [file pone.0080138.s005.doc]

Supplemental Table 5. Comparison of spiral ganglion cell density (cells/mm^2^) 21 days after blast exposure.

| Turn of spiral ganglion | NC | B | B/T | *F* value | *p* value |
| --- | --- | --- | --- | --- | --- |
| Basal | 462.58 ± 43.39 | 457.76 ± 47.16 | 469.24 ± 35.81 | (2, 37) = 0.02 | > 0.05 |
| Middle | 681.88 ± 22.92 | 722.77 ± 44.92 | 770.99 ± 22.37 | (2, 59) = 1.91 | > 0.05 |
